# Characteristics of Patients With Pulmonary Arterial Hypertension in a Pulmonary Hypertension Association-Accredited Comprehensive Care Center: A Contrast in Features When Compared With US National Registry Data

**DOI:** 10.7759/cureus.31764

**Published:** 2022-11-21

**Authors:** Dominique Ingram, Ayedh K Alamri, Brittany A Penn, Jennalyn D Mayeux, Christy L Ma, Katharine R Clapham, Anu E Abraham, Dana Klanderud, Ben Sadeh, Emily M Beck, Nathan D Hatton, John J Ryan

**Affiliations:** 1 Cardiovascular Medicine, University of Utah, Salt Lake City, USA; 2 Department of Medicine, College of Medicine, Northern Border University, Arar, SAU; 3 Pulmonary and Critical Care Medicine, University of Utah, Salt Lake City, USA; 4 Cardiovascular Medicine, University of Utah, Salt Lake City, UT, USA; 5 Pulmonary and Critical Care Medicine, George E. Wahlen Department of Veterans Affairs Medical Center, Salt Lake City, USA

**Keywords:** connective tissue disorder (ctd), idiopathic pulmonary arterial hypertension, s: epidemiology, methamphetamine induced pulmonary hypertension, – pulmonary hypertension

## Abstract

Background

Since the initial description in the 1980s, our understanding of the diversity of pulmonary arterial hypertension (PAH) has continued to evolve. In this study, we report the characteristics of patients seen in an academic medical center for PAH from August 2020 through November 2021 and contrast those with nationally reported data from the United States Pulmonary Hypertension Scientific Registry (USPHSR).

Study Design

Investigators at the University of Utah Pulmonary Hypertension Program prospectively enrolled adult patients diagnosed with WHO Group 1 PAH, who were evaluated between August 2020 and November 2021 in a program-specific registry. Patient exposure and health histories were collected through structured interviews and questionnaires, along with clinical data and medication use. A total of 242 patients were enrolled in the University of Utah Pulmonary Hypertension Registry (UUPHR).

Results

Of the 242 enrolled patients, the most common etiology was associated PAH (APAH), accounting for 71.1% of the population. The second largest etiology was idiopathic PAH (IPAH) at 26.4%. The remaining patients were distributed between familial PAH (FPAH), pulmonary veno-occlusive disease (PVOD), and others. Of the total population classified as APAH, 39% of cases were noted as secondary to connective tissue disease (CTD) and 33% as toxin-induced. These represented 28% and 24% of the total population, respectively.

Conclusions

In this US-based accredited academic medical center, the etiology of PAH in our patient population contrasts with national registry data. In the UUPHR, APAH, specifically CTD-PAH and toxin-associated PAH, accounts for the majority of patients with PAH. This contrasts with IPAH, which nationally is the most reported cause of PAH. Differences in our population may reflect the regional variation of the referral site, but it is noteworthy for its contrast with historically reported phenotypes.

## Introduction

Pulmonary arterial hypertension (PAH) is a rare disease characterized by increased mean pulmonary artery pressure with resultant right ventricular dysfunction and, ultimately, failure. The current three-year mortality rate remains significant at 21% [1-2]. Risk factors that contribute to the development of PAH include connective tissue disease (CTD), liver disease, hereditary gene mutations, congenital heart disease (CHD), HIV, hereditary hemorrhagic telangiectasia (HHT), schistosomiasis, and various toxin exposures. A significant number of cases are labeled as idiopathic. Prognosis varies depending on the underlying etiology, and in turn, treatment options may differ because of the varying levels of risk within each phenotype. Deaño RC et al. showed that 33% of patients with a diagnosis of PAH are misdiagnosed due to the lack of a thorough diagnostic evaluation and work-up [3]. We hypothesize that the common failure to perform a good work-up, including inadequately assessing PAH risk factors, could contribute to the large number of patients who are defined as idiopathic PAH (IPAH). In this study, we enrolled adults diagnosed with WHO Group 1 PAH seen by the University of Utah Pulmonary Hypertension Comprehensive Care Center between August 2020 and November 2021 in a program-specific registry. PAH-risk factors were systematically collected and recorded, including toxin exposure history and diagnostic data. A total of 242 patients were enrolled in the University of Utah Pulmonary Hypertension Registry (UUPHR). This data was previously presented at the PHA 2022 International PH Conference and Scientific Sessions in June of 2022.

## Materials and methods

Adult patients evaluated in the University of Utah Pulmonary Hypertension Comprehensive Care Center between August 2020 and November 2021 were enrolled in UUPHR. Criteria for enrollment included a prior diagnosis using criteria similar to the Registry to Evaluate Early and Long-term PAH Disease Management (REVEAL Registry) registry or a new diagnosis of PAH based on current guidelines [4-6]. The hemodynamic criteria used were a mean pulmonary artery pressure (mPAP) greater than or equal to 20 mmHg and pulmonary capillary wedge pressure (PCWP) of less than or equal to 18 mmHg. A total of 242 patients were enrolled. This study was approved by institutional IRB.

The diagnosis was determined through a structured interview for comprehensive health history, including personal and family health history, drug and toxin exposures, medical record review, and diagnostic testing. Testing included, but was not limited to, right heart catheterization (RHC), ECG, pulmonary function testing (PFT), six-minute walk distance (6MWD), ventilation and perfusion scan (VQ), chest imaging, sleep studies, standardized toxicology screening, and serum studies. Enrolled patients were classified into subsets of PAH based on the above data.

Demographic and clinical information was collected on each patient, including age, sex, race, BMI, New York Heart Association (NYHA) functional class, 6MWD, and hemodynamics from RHC. In addition, medication use, including the use of PAH-specific therapies, was recorded. Oral PAH therapies included calcium channel blockers (CCB), phosphodiesterase-5 inhibitors (PDE5I: sildenafil or tadalafil), endothelin receptor antagonists (ERA: ambrisentan or macitentan), soluble guanylate cyclase stimulator (sGCS: riociguat), and prostanoids (selexipag and treprostinil). Other agents in the prostacyclin pathways included inhaled treprostinil and epoprostenol, subcutaneous treprostinil, and intravenous treprostinil and epoprostenol. Combined therapies were separated into three groups: combination, oral included an ERA and either PDE5I or sGCS; combination, oral/prostacyclin included one of the oral therapies and one of the prostacyclin pathways; and combination, triple therapy indicated the use of PDE5I or sGCS, an ERA and one prostacyclin pathway.

Patients were classified based on the underlying etiology of PAH: IPAH, familial or heritable PAH (FPAH), pulmonary veno-occlusive disease (PVOD), and associated forms of PAH (APAH). The APAH population was further characterized into subsets based on the specific etiology. APAH subgroups include CTD, porto-pulmonary hypertension (PoPH), CHD, and toxin-APAH. There were few patients with PAH secondary to HIV, HHT, and hematologic malignancies, and these subtypes were combined and classified as other APAH. There were no patients in our registry that were felt to have a schistosomiasis-related disease.

Descriptive statistics were used to define the characteristics of the population. Quantitative variables were expressed as mean and SD, and qualitative variables as count and percentage. These were performed using Microsoft Excel. This data was compared to that reported by the United States Pulmonary Hypertension Scientific Registry (USPHSR) [7]. Unpaired t-tests were used to compare the mean, SD, and sample size (n) between the UUPHR and USPHSR. A p-value of <0.05 was considered significant. The statistical analyses were performed using Prism by Graph Pad.

## Results

**Figure 1:**
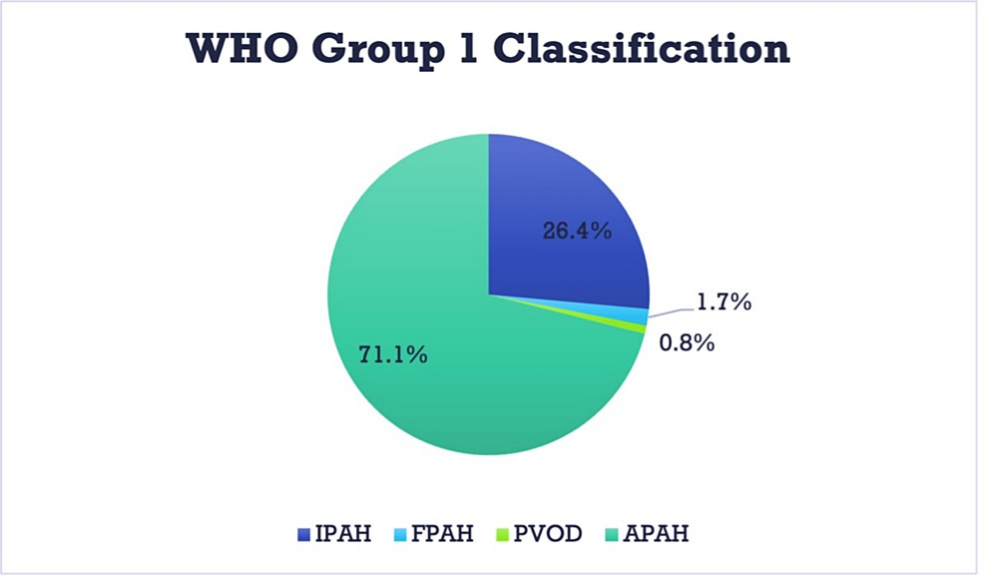
Distribution by subset of group 1 PAH. IPAH: Idiopathic pulmonary arterial hypertension; FPAH: Familial pulmonary arterial hypertension; PVOD: Pulmonary veno-occlusive disease; APAH: Associated pulmonary arterial hypertension.

Within a 15-month period, 242 patients with a diagnosis of WHO group 1 PAH were evaluated and enrolled in UUPHR. The most frequent subset was APAH (71.1%). The second most common subset was IPAH (26.4%). The remaining population was distributed between FPAH and PVOD (Figure 1). 

**Figure 2:**
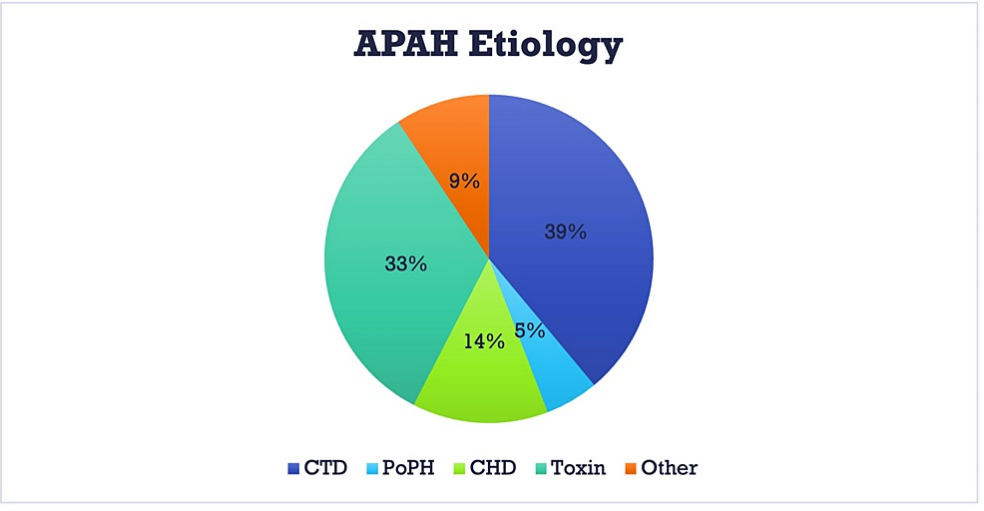
Distribution of subgroups of APAH. CTD: Connective tissue disease; PoPH: Porto-pulmonary hypertension; CHD: Congenital heart disease; Other: HIV, HHT, hematologic malignancy; APAH: Associated pulmonary arterial hypertension.

Of the APAH subtypes, CTD (39%) and toxin-associated (33%) were the most common and represented 28% and 24% of the overall population, respectively (Figure 2).

Of the total population, 71.9% were female, the average age was 58, and 11.6% were over 75 (Table 1). Of the APAH population, the subgroup with the largest percentage of patients over the age of 75 was CTD at 14.9%. CTD also had the largest proportion of female patients (91%) (Table 2).

**Table 1:** Demographics and characteristics of all enrolled subjects according to WHO Group 1. Listed as No. (%) or average ± SD unless otherwise indicated. IPAH: Idiopathic pulmonary arterial hypertension; FPAH: Familial pulmonary arterial hypertension; PVOD: Pulmonary veno-occlusive disease; APAH: Associated pulmonary arterial hypertension.

Characteristic	All Patients	IPAH	FPAH	PVOD	APAH	P-value
Patients	242	64	4	2	172	
Age Group						0.024
25-64 y	148 (61.2)	33 (51.6)	3 (75.0)	2	110 (64.0)	
65-74 y	59 (24.4)	16 (25.0)	1 (25.0)	0	43 (25.0)	-
> 75 y	28 (11.6)	16 (25.0)	0	0	12 (7.0)	-
Deceased	7 (2.9)	0	0	0	7 (4.1)	-
Female sex	174 (71.9)	52 (81.3)	3 (75.0)	1 (50.0)	119 (69.2)	-
Race						
Asian	4 (1.7)	1 (1.6)	0	0	3 (1.7)	-
Black	6 (2.5)	0	0	0	6 (3.5)	-
Native American/Alaskan	5 (2.1)	1(1.6)	0	0	4 (2.3)	-
Pacific Islander	2 (0.8)	0	1(25.0)	0	1 (0.6)	-
White	208 (86.0)	57 (89.1)	3 (75.0)	2	146 (84.9)	-
Other	17 (7.0)	5 (7.8)	0	0	12 (7.0)	-
Hispanic or Latino ethnicity	19 (7.9)	7 (10.9)	0	0	12 (7.0)	-
BMI, kg/m²	30 ± 7.18	31.82 ± 6.85	31.81 ± 9.21	34	29.21 ± 7.18	0.156

**Table 2:** Demographics and characteristics organized by APAH subgroup. Listed as N (%) or average ± SD unless otherwise indicated. CTD: Connective tissue disease; PoPH: Porto-pulmonary hypertension; CHD: Congenital heart disease; APAH: Associated pulmonary arterial hypertension.

Characteristic	CTD	PoPH	CHD	Toxin-Associated	Other
Patients	67	9	23	57	16
Age Group					
25-64 y	31 (46.2)	5 (55.6)	17 (73.9)	51 (89.5)	9 (56.3)
65-74 y	21 (31.3)	4 (44.4)	5 (21.7)	9 (15.8)	5 (31.3)
> 75 y	10 (14.9)	0	1 (4.3)	1 (1.8)	0
Deceased	5 (7.5)	0	1 (4.3)	0	1 (6.3)
Female sex	61 (91.0)	5 (55.6)	12 (52.2)	31 (54.5)	10 (62.5)
Race					
Asian	2 (3.0)	0	0	1 (1.8)	0
Black	4 (6.0)	0	1 (4.3)	1 (1.8)	0
Native American/Alaskan	4 (6.0)	0	0	0	0
Pacific Islander	0	0	0	0	1 (6.3)
White	51 (76.1)	8 (88.9)	21 (91.3)	53 (93.0)	15 (93.8)
Other	6 (9.0)	1 (11.1)	2 (8.7)	2 (3.5)	0
Hispanic or Latino ethnicity	6 (9.0)	1 (11.1)	1 (4.3)	3 (5.3)	0
BMI, kg/m²	27.77 ± 6.42	30.43 ± 10.86	30.90 ± 5.87	29.49 ± 6.75	31.65 ± 10.19

The diagnostic data showed that the total population had an average mPAP of 41.46 mmHg (SD 14.35 mmHg), an average PCWP of 11.09 mmHg (SD 3.37 mmHg), and an average 6WMD of 405.95 meters (SD 137.45 meters). The most common NYHA functional class was II at 56.5%, followed by III at 26.0% (Table 3). The group with the highest average mPAP was toxin-associated at 47.42 mmHg (SD 14.34), and the lowest was PoPH at 36.89 mmHg (SD 8.54) (Table 4).

**Table 3:** Diagnostic data including functional class, 6MWD, right heart catheterization according to WHO Group 1. *PVR was calculated by thermodilution unless cardiac output was only measured by Fick; Listed as N (%) or average ± SD unless otherwise indicated. IPAH: Idiopathic pulmonary arterial hypertension; FPAH: Familial pulmonary arterial hypertension; PVOD: Pulmonary veno-occlusive disease; 6MWD: Six-minute walk distance; mPAP: Mean pulmonary artery pressure; PCWP: Pulmonary capillary wedge pressure; PVR: Pulmonary vascular resistance; APAH: Associated pulmonary arterial hypertension.

Characteristic	All Patients	IPAH	FPAH	PVOD	APAH	P-value
Functional class	n = 242	n = 64	n = 4	n = 2	n = 172	-
I	10 (4.1)	4 (6.3)	0	0	6 (3.5)	-
II	144 (59.5)	44 (68.8)	1 (25)	0	99 (57.6)	-
III	63 (26.0)	12 (18.8)	2 (50)	2	47 (27.3)	-
IV	8 (3.3)	0	0	0	8 (4.7)	-
Not available	17 (7.0)	4 (6.3)	1 (25)	0	12 (47.0)	-
6MWD, m	405.95 ± 137.45	390.57 ± 135.23	361.67 ± 124.85	438	412.46 ± 139.22	<0.001
N	201	54	3	1	143	-
mPAP, mmHg	41.46 ± 14.35	40.63 ± 14.05	46.5 ± 19.28	42.5	41.65 ± 14.49	<0.001
N	242	64	4	2	172	-
PCWP, mmHg	11.09 ± 3.37	10.92 ± 3.04	13.5 ± 1.29	11.5	11.09 ± 3.53	0.001
N	242	64	4	2	172	-
mRAP, mmHg	8.75 ± 4.50	8.81 ± 3.87	12.25 ± 2.5	5	8.68 ± 4.72	0.155
N	239	59	4	1	164	-
PVR, wu*	7.65 ± 6.43	6.87 ± 4.57	8.37 ± 5.82	4.84	7.94 ± 7.02	<0.001
N	238	61	4	2	171	-
CO, TD	5.14 ± 1.61	5.19 ± 1.43	4.81 ± 1.09	6.93	5.12 ± 1.68	-
N	205	53	3	1	149	-
CI, TD	2.73 ± 0.80	2.61 ± 0.62	2.59 ± 0.45	3.37	2.77 ± 0.86	-
N	194	51	3	1	139	-
CO, Fick	5.06 ± 1.62	5.01 ± 1.77	3.4	6	5.11 ± 1.63	-
N	32	9	1	1	21	-
CI, Fick	2.49 ± 0.73	2.47 ± 0.63	1.74	3.21	2.46 ± 0.73	-
N	29	7	1	1	26	-

**Table 4:** Diagnostic data including functional Class, 6MWD, right heart catheterization by APAH subgroup. Listed as N (%) or average ± SD, unless otherwise indicated. CTD: Connective tissue disease; PoPH: Porto-pulmonary hypertension; CHD: Congenital heart disease; 6MWD: Six-minute walk distance; mPAP: Mean pulmonary artery pressure; PCWP: Pulmonary capillary wedge pressure; PVR: Pulmonary vascular resistance; APAH: Associated pulmonary arterial hypertension.

Characteristics	CTD	PoPH	CHD	Toxin-Associated	Other
Functional class	n = 67	n = 9	n = 23	n = 57	n = 16
I	2 (3.0)	1 (11.1)	0	2 (3.5)	1 (6.3)
II	42 (62.7)	3 (33.3)	16 (69.6)	28 (49.1)	10 (62.5)
III	18 (26.9)	3 (33.3)	3 (13.0)	19 (33.3)	4 (25.0)
IV	3 (4.5)	1 (11.1)	1 (4.3)	3 (5.3)	0
Not available	2 (3.0)	1 (11.1)	3(13.0)	5 (8.8)	1 (6.3)
6MWD, m	379.66 ± 160.10	394.63 ± 130.98	453.63 ± 128.57	443.57 ± 118.03	395.79 ± 109.02
N	56	8	19	46	14
mPAP, mmHg	36.99 ± 13.26	36.89 ± 8.54	41.22 ± 16.46	47.42 ± 14.34	43.88 ± 13.19
N	67	9	23	57	16
PCWP, mmHg	10.85 ± 3.63	10.89 ± 4.26	12.70 ± 3.42	10.77 ± 3.44	11.06 ± 2.93
N	67	9	23	57	16
mRAP, mmHg	7.75 ± 4.71	6.25 ± 3.33	9.14 ± 3.78	9.64 ± 5.01	9.63 ± 4.87
N	63	8	22	55	16
PVR, wu*	6.53 ± 5.96	5.17 ± 3.53	5.80 ± 5.05	10.23± 6.69	8.53 ± 8.12
N	67	9	23	57	15
CO, TD	5.04 ± 1.33	6.29 ± 2.81	5.42 ± 1.59	4.80 ± 1.70	5.60 ± 2.14
N	61	8	17	49	13
CI, TD	2.90 ± 0.78	3.44 ± 1.25	2.72 ± 0.78	2.47 ± 0.78	2.94 ± 1.06
N	58	7	16	46	13
CO, Fick	5.4 ± 1.73	NA	6.38 ± 1.26	4.21 ± 1.24	4.69
N	6	NA	5	8	2
CI, Fick	2.69 ± 0.79	NA	2.87 ± 0.77	2.29 ± 0.80	2.14
N	6	NA	5	8	2

Nearly 98% of the population were on at least one PAH-specific therapy. Of those, 34% were on monotherapy, 33% were on a combination of dual oral therapy with ERA and PDE5I or sGCS, 38% were on a combination of one oral therapy and one prostacyclin pathway, and 26% were on a combination of two oral therapies and a prostacyclin pathway referred to as triple combination therapy (Table 5).

**Table 5:** PAH-specific medications among patients. Combination, oral therapy: one PDE5i or riociguat and one ERA; Combination oral/prostacyclin: one oral therapy (PDE5i, ERA, riociguat) and one prostacyclin pathway; Combination, triple: one PDE5i or riociguat, one ERA, or prostacyclin pathway. CCB:  Calcium channel blockers; PDE: Phosphodiesterase; ERA: Endothelin receptor antagonists.

Variable		Oral Therapy				Prostacyclin Pathway			
	CCB	PDE-5 Inhibitor	ERA	Riociguat	Selexipag	Oral Treprostinil	Inhaled Treprostinil	Subcutaneous Treprostinil	IV Treprostinil
Overall, N=237	38 (16)	218 (92)	146 (62)	8 (3)	40 (17)	2 (<1)	3 (1)	22 (9)	5 (2)
Monotherapy, N=81	0	74 (34)	6 (4)	1 (13)	0	0	0	0	0
Combination, Oral Therapy, N=79	0	76 (35)	79 (54)	3 (37)	0	0	0	0	0
Combination, Oral/Prostacyclin, N=11	0	11 (5)	0	0	6 (15)	0	0	4 (18)	1 (25)
Combination, Triple, N=61	0	57 (26)	61 (42)	4 (50)	34 (85)	2	3	18 (82)	4 (75)

## Discussion

The data collected in UUPHR contrasts with national registry data published from USPHSR in 2021. In the UUPHR population, only 26.4% were IPAH, while 71.1% had APAH. This differs significantly from the percentages reported by the USPHSR registry (44% IPAH and 51% APAH). We believe that toxin-associated PAH is under-represented in national registry data. In the Utah registry, 24% of the patients had toxin-APAH, while this accounted for only 4.8% of patients in USPHSR. Of note, 89.5% of the toxin-APAH was reported as secondary to methamphetamine usage and comprised 21.5% of the total population of patients in the UUPHR. This contrasts with the reported 4.5% of the USPHSR. The lack of nationally accepted standardized drug screening protocols and the established prevalence of methamphetamines in the western US may contribute to these differences, but further investigation is warranted [8].

Our registry data was similar to the UUPHSR in regard to demographic information and incidence of CTD-APAH. UUPHR reports that the most common demographic is females, with an average age of 58. This is consistent with USPHSR's reported average age of 55.8. UUPHR does have a larger overall percentage of older individuals compared to the national registry, with 11.6% of the population being 75 years of age or older, whereas the USPHSR reports 4.4%. However, the numbers reported from UUPHR are consistent with data from the French PH registry, where similarly, approximately 10% of patients were over 75 [9]. Regarding CTD, the Utah registry was similar to the USPHSR, with 28% of the total patients classified as CTD-associated PAH (34% in USPHSR).

The data show that our center's management of PAH is consistent with the nationally reported use of therapies. However, while our center reports that 26% of patients were on triple PAH therapy, USPHSR reports 15%. In addition, our center reports the most frequent NYHA functional class as II (59.5%), while USPHR reports the most common as III (45.2%). These discrepancies may reflect the benefit of earlier initiation of combination therapy [10-15].

Limitations

There are several limitations to this study. Our program encompasses a geographically large and rural catchment area with regional variations in access to care, patient demographics, and social determinants of health where substance use may be more common than in other areas of the country. Our patients are not routinely genotyped due to financial barriers within the United States, so the prevalence of FPAH is poorly defined. Some patients were started on PAH-directed therapies before enrollment and may lack complete data sets from external referral centers. Due to the revised PAH hemodynamic definition and lack of complete data sets from external referral centers, enrollment criteria were softened to align with the REVEAL registry. As such, we allowed patients with a PCWP up to 18 mmHg, and some patients with a prior diagnosis had a pulmonary vascular resistance (PVR) of <3 Wood Units (WU). Thus, the specificity for inclusion was reduced.

## Conclusions

In this US-based accredited academic medical center registry, the subsets of PAH contrast with national registry data. In the UUPHR, APAH accounts for most patients with PAH, with a significant portion of the population classified as toxin-associated PAH. Nationally, IPAH is the most reported cause of PAH. Differences in our population may reflect the regional variation of the referral site, but it is noteworthy for its contrast with historically reported phenotypes. An increased publication of regional registry data is required to accurately reflect regional variations in the burden of subclasses of PAH.
